# Bivariate linear mixed model analysis to test joint associations of genetic variants on systolic and diastolic blood pressure

**DOI:** 10.1186/1753-6561-8-S1-S75

**Published:** 2014-06-17

**Authors:** Binod Neupane, Joseph Beyene

**Affiliations:** 1 Population Genomics Program, McMaster University, 1200 Main Street West, Hamilton, Ontario, L8N 3Z5, Canada

## Abstract

Genetic variants that predispose adults and the elderly to high blood pressure are largely unknown. We used a bivariate linear mixed model approach to jointly test the associations of common single-nucleotide polymorphisms with systolic and diastolic blood pressure using data from a genome-wide association study consisting of genetic variants from chromosomes 3 and 9 and longitudinal measured phenotypes and environment variables from unrelated individuals of Mexican American ethnicity provided by the Genetic Analysis Workshop 18. Despite the small sample size of a maximum of 131 unrelated subjects, a few single-nucleotide polymorphisms appeared significant at the genome-wide level. Simulated data, which was also provided by Genetic Analysis Workshop 18 organizers, showed higher power of the bivariate approach over univariate analysis to detect the association of a selected single-nucleotide polymorphism with modest effect. This suggests that the bivariate approach to longitudinal data of jointly measured and correlated phenotypes can be a useful strategy to identify candidate single-nucleotide polymorphisms that deserve further investigation.

## Background

High blood pressure is a common disorder in adults and the elderly and is associated with increased risk of cardiovascular diseases and many other morbidities [1]. It is a complex trait that can be influenced by certain genetic makeups, environmental factors, or their interactions. There have been some genetic association studies to identify the effect of genetic component on blood pressure in certain ethnic population. The San Antonio Family Studies (SAFS) is a family-based longitudinal study designed to identify genes associated with high blood pressure in Mexican American population. For Genetic Analysis Workshop 18 (GAW18), the data from SAFS was provided for odd-numbered chromosomes.

In multivariate longitudinal data, multiple response variables are jointly measured over time from the same individuals. Other environmental variables can also change over time and could have been recorded. To understand the relationship between independent and multivariate dependent response variables, we need to take into account the correlation between multivariate responses measured over time. In GAW18 data, the phenotypic data, namely systolic blood pressure (SBP) and diastolic blood pressure (DBP), as well as some environmental data, were measured over time and these phenotypic data from the same individuals are likely to be correlated as they might be regulated by the same genes or common environments. Therefore, we used a bivariate linear mixed-effect model approach to test the association of genetic variants with bivariate phenotypic values adjusting for environmental factors.

## Methods

### Participants, genotyping data, and quality control

Details about sample recruitment can be found in Hunt et al [2]. For GAW18, data from  959 participants of Mexican American ethnicity from 20 large pedigrees, who were followed up for up to 4 times from 1991 to 2011, were provided. However, for our analysis we considered only 157 unrelated members from this cohort, of whom only 142 individuals had both genotypic and phenotypic data.

We analyzed genome-wide association studies (GWAS) data from chromosomes 3 and 9. There were 65,519 single-nucleotide polymorphisms (SNPs) on chromosome 3 and 42,177 SNPs on chromosome 9. We restricted our analysis to common SNPs (minor allele frequency ≥0.05) with genotyping call rates ≥0.95 and Hardy-Weinberg equilibrium *p *value ≥10^−3 ^and subjects with genotyping call rates ≥0.95. We applied these criteria to those 142 individuals using PLINK.

### Statistical method

We applied a bivariate linear mixed model framework (more details can be found in Refs. [3,4]) in order to test for association between individual common genetic variant with SBP and DBP jointly. For trait *k*(*k *= 1,2), suppose $Y_{i}^{k}$ is a (*n_i _*× 1) vector of the trait values for $n_{i}$ times of measurements for the subject *i*$\left(i=1,2,\ldots ,N\right)$; then, the univariate mixed-effect model with *p *independent variables with $q\left(q\leq p\right)$ of them having random effects, can be expressed as [3,4]

(1) $$Y_{i}^{k}=X_{i}^{k}\beta^{k}+Z_{i}^{k}\gamma_{i}^{k}+W_{i}^{k}+\varepsilon_{i}^{k}$$

where $X_{i}^{k}$ is a $\left(n_{i}\times p\right)$ design matrix that results in the systematic variation in the *k*^th ^trait with $\beta^{k}$ as the corresponding (p × 1) vector of fixed-effect; $Z_{i}^{k}$ is a (*n_i _*× *q*) design matrix, usually a subset of $X_{i}^{k}$(q≤p) that characterize the random variation in the trait with $\gamma_{i}^{k}\sim N\left(0,G^{k}\right)$ as the corresponding $\left(q\times 1\right)$ vector of random effect; $W_{i}^{k}\sim N\left(0,R_{i}^{k}\right)$ is a (*n_i _*× 1) vector of the stochastic processes (within subject errors over repeated times) with realization $w_{i}^{k}\left(t\right)$ at time *t* with variance $R_{i}^{k}\left(t\right)=\sigma_{w^{k}}^{2}$ and covariance $R_{i}^{k}\left(s,t\right)=cov\left(w_{i}^{k}\left(s\right),w_{i}^{k}\left(t\right)\right)=\sigma_{w^{k}}^{2}e^{\lambda^{k}\left(t-s\right)}$ at times *s *and *t*, 0 ≤s<t; and $\varepsilon_{i}^{k}\sim N\left(0,\sigma_{\varepsilon^{k}}^{2}I_{n_{i}}\right)$ is a (*n_i _*× 1) vector of random errors, where $I_{n_{i}}$ is an identity matrix. If the 2 traits $Y_{i}^{1}$ and $Y_{i}^{2}$ are correlated, then bivariate linear mixed model can be formulated as

(2) $$\left[\begin{array}{c} Y_{i}^{1}\\ Y_{i}^{2} \end{array}\right]=\left[\begin{array}{cc} X_{i}^{1} & 0\\ 0 & X_{i}^{2} \end{array}\right]\;\left[\begin{array}{c} \beta^{1}\\ \beta^{2} \end{array}\right]+\left[\begin{array}{cc} Z_{i}^{1} & 0\\ 0 & Z_{i}^{2} \end{array}\right]\;\left[\begin{array}{c} \gamma_{i}^{1}\\ \gamma_{i}^{2} \end{array}\right]+\left[\begin{array}{c} \varepsilon_{i}^{1}\\ \varepsilon_{i}^{2} \end{array}\right]$$

That is, $Y_{i}=X_{i}\beta +Z_{i}\gamma_{i}+W_{i}+\varepsilon_{i}$, where, $\gamma_{i}\sim N\left(0,G\right);W_{i}\sim N\left(0,R_{i}\right);\epsilon_{i}\sim N\left(0,{\text{Σ}}_{i}\right);$

$G=\left(\begin{array}{cc} G^{1} & G^{12}\\ G^{12} & G^{2} \end{array}\right);{\text{Σ}}_{i}=\text{Σ}⊗I_{n_{i}};\text{Σ}=\left(\begin{array}{cc} \sigma_{\epsilon 1}^{2} & 0\\ 0 & \sigma_{\epsilon 2}^{2} \end{array}\right)$. Here, ⊗ is the Kronecker product. The $W_{i}$ is the bivariate stochastic processes that not only captures the correlation of measurements within the same subject at multiple times, but also the correlation between 2 traits at the same time for the subject, and has the variance matrix $R_{i}\left(t\right)=C=\left(\begin{array}{cc} \sigma_{w^{1}}^{2} & \sigma_{w^{1}w^{2}}\\ \sigma_{w^{1}w^{2}} & \sigma_{w^{2}}^{2} \end{array}\right)$ at time *t *and covariance matrix $R_{i}\left(s,t\right)=Ce^{B\left(t-s\right)}$ at times *t *and *s*, 0≤s<t. Two traits are independent if $\sigma_{w^{1}w^{2}}=0$. Here, *B *is a 2×2 real matrix chosen such that the eigenvalues of *B *have negative real parts and matrices *C *and $--\left(CB+B^{\prime }C\right)$ are positive semidefinite symmetric [3]. We have $E\left(Y_{i}\right)=X_{i}\beta$ and $var\left(Y_{i}\right)=Z_{i}G_{i}Z_{i}^{\prime }+R_{i}+{\text{Σ}}_{i}$ under independence assumption of $\gamma_{i},W_{i}$ and $\varepsilon_{i}$; thus $Y_{i}\sim N\left(X_{i}\beta ,Z_{i}G_{i}Z_{i}^{\prime }+R_{i}+{\text{Σ}}_{i}\right).$ Solution for $\beta ={\left(\beta^{1},\beta^{2}\right)}^{\prime }$ can be obtain by maximum likelihood or restricted maximum likelihood (REML) approach using multivariate normal likelihood of $Y_{i}.$

### Data analysis

After the quality control filtering, we had only 133 participants available for bivariate analysis, who had genotype data and at least 1 measurement of phenotypic data. There were 52,862 SNPs from chromosome 3 and 34,475 SNPs from chromosome 9 that passed the filtering criteria.

For the bivariate linear mixed-effect model fitting for each SNP, genotype (0, 1, or 2 for the number of copies of minor allele) of a SNP was the independent variable of interest. Besides, we a priori selected to include 3 covariates, namely, measurement time in years at an examination since enrollment (which is 0 for the first year of enrollment), baseline age (at enrollment), and repeatedly measured antihypertensive medication use in the model. We also included sex and repeatedly measured smoking status in the model because keeping them, each separately or together, resulted in a better fit (smaller Akaike information criteria [AIC]) of the bivariate model for the 20 SNPs from chromosome 9 selected for model fit assessment (modeling techniques description given in a successive paragraph). We considered autoregressive order-1 (AR(1)) assumption used for repeated measured analysis and unstructured (UN) variance components used for the random-effect analysis to identify the appropriate covariance (or correlation) structure for between and within the 2 phenotypic measurements over time [4,5]. The AR(1) assumption did not lead to the noticeably improved model fit but did involve some unrealistic assumptions in bivariate modeling, such as measurement at equal interval of time for both phenotype at a time and for a phenotype over time. Therefore, we assumed unstructured variance components assumption that allows correlations between any 2 measurements for the same phenotype and between 2 phenotypes at a time to vary across subjects.

There were missing follow-up data on blood pressure and other repeatedly measured covariates, where data from 97 subjects were missing in the fourth enrollment period. The available data for the fourth period were not used because discarding them resulted in much better fit for each of those 20 SNPs. Next, 2 subjects had missing data on medication use and smoking in all 3 examinations. Thus, the effective sample size was 131 for real data analysis. No attempt was made to use imputation.

A binary variable "BPTYPE" (blood pressure type) was defined as BPTYPE = 1 for SBP and = 0 for DBP for a subject at a measurement time. Finally, equation (2) was fitted for each SNP, where the repeatedly measured bivariate blood pressure (a maximum of six possible measurements for two phenotypes at three examinations for a subject) was regressed against genotype and the covariates specified above (each regressor was multiplied by BPTYPE in order to perform bivariate analysis) using MIXED procedure in SAS (see Refs. [4,5] for details of modeling technique and SAS codes). In the analysis using REML, we considered BPTYPE having group effect, patients' ID having subject effects, and measurement time having random effect; that is, time effects on blood pressure varies across individuals.

For the genotype effects $\beta_{G}={\left(\beta_{1}=\beta_{G}^{SBP},\beta_{2}=\beta_{G}^{DBP}\right)}^{\prime }$ on SBP and DBP, we estimated ${\hat{\beta }}_{G}={\left({\hat{\beta }}_{1},{\hat{\beta }}_{2}\right)}^{\prime }$ and the corresponding covariance matrix $S=\left(\begin{array}{cc} s_{1}^{2} & s_{12}\\ s_{12} & s_{2}^{2} \end{array}\right)$, $s_{1}$ and $s_{2}$ being standard errors of ${\hat{\beta }}_{1}$ and ${\hat{\beta }}_{2}$, respectively, and $s_{12}$ their covariance, per 1 copy increase in minor allele of each SNP, using the bivariate approach. For each SNP, we tested the null hypothesis ${\text{H}}_{0}:\beta =0$*vs*. alternative ${\text{H}}_{1}:\beta \neq 0$ (ie H_1 _: at least1, $\beta_{k}\neq 0;\text{k}=1,2$; that is, the genotype was associated with at least 1 phenotype) using F-test with $\left(\vartheta_{1}=2,\vartheta_{2}=360\right),$ degrees of freedom [6], assuming multivariate normality of ${\hat{\beta }}_{G}$. We also tested the hypothesis with $\chi_{2}^{2}$ test statistic assuming large sample approximation [6]. Because the data arose from GWAS, we used a genome-wide significance threshold, *α *= 7.2 × 10^−8 ^[7], to adjust for multiple testing problems, which enables us to see if any SNPs from chromosome 3 or 9 achieve this threshold in bivariate analysis.

### Simulation

We assessed the statistical power of the bivariate linear mixed effect model using 200 simulated longitudinal data sets provided by GAW18 organizers. However, we considered the data from only 142 unrelated subjects who had genotypic data and phenotype and covariate information from all 3 examinations. We chose to assess power to detect the association of a common SNP, *rs6442089 *(from MAP4 gene). The SNP had the effect sizes, *β*_1 _= −1.4951 (variation explained =0.0117%) and *β*_2 _= −2.3810 (variation explained = 0.0143%) in simulated SBP and DBP, respectively, per copy increase in minor allele. We used data from 141 subjects as genotype data was missing for the SNP in 1 subject. We employed the same regression analysis model and modeling technique, and assessed the same hypothesis using F $\left(\vartheta_{1}=2,\vartheta_{2}=552\right)$ and $\chi_{2}^{2}$ test statistics as in real data analysis above. Power of univariate linear mixed model analysis to detect the effect of the same SNP separately on SBP and DBP . $\left(H_{0}:\beta_{k}=0\;vs\;H_{1}:\beta_{k}\neq 0,k=1,2\right)$ was also assessed using the same regression model and assumption as in bivariate case using F $\left(\vartheta_{1}=1,\vartheta_{2}=137\right)$ and $\chi_{1}^{2}$ test statistics. We assessed the power at α=0.05 as there was no issue of multiple testing; however, we also used *α *= 7.2 × 10^−8 ^to be consistent with real data analysis.

## Results

### Real data analysis

In our sample data, the mean age (standard deviation) at enrollment was 53.7 (16.0) years, where subjects were 20.3 to 94.2 years old when enrolled. In a graphical inspection, the SBP and DBP data at each and all 3 examinations looked approximately normal.

**Figure 1:**
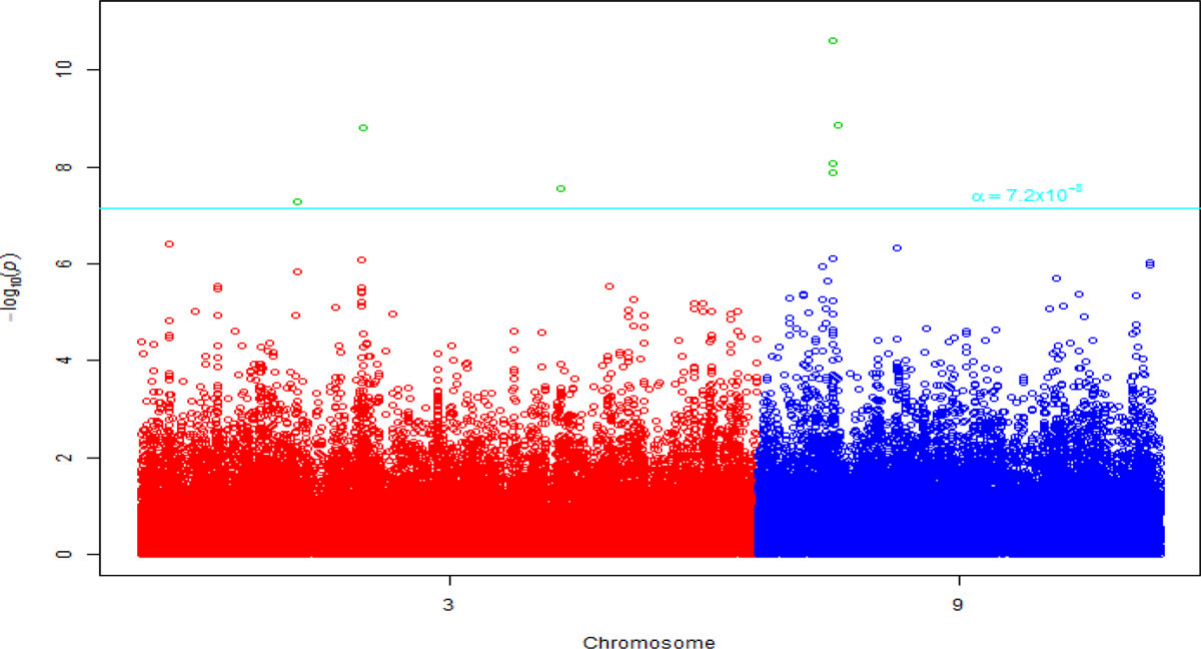
**Manhattan plot of joint association *p *values for variants on chromosomes 3 and 9**.

**Figure 2:**
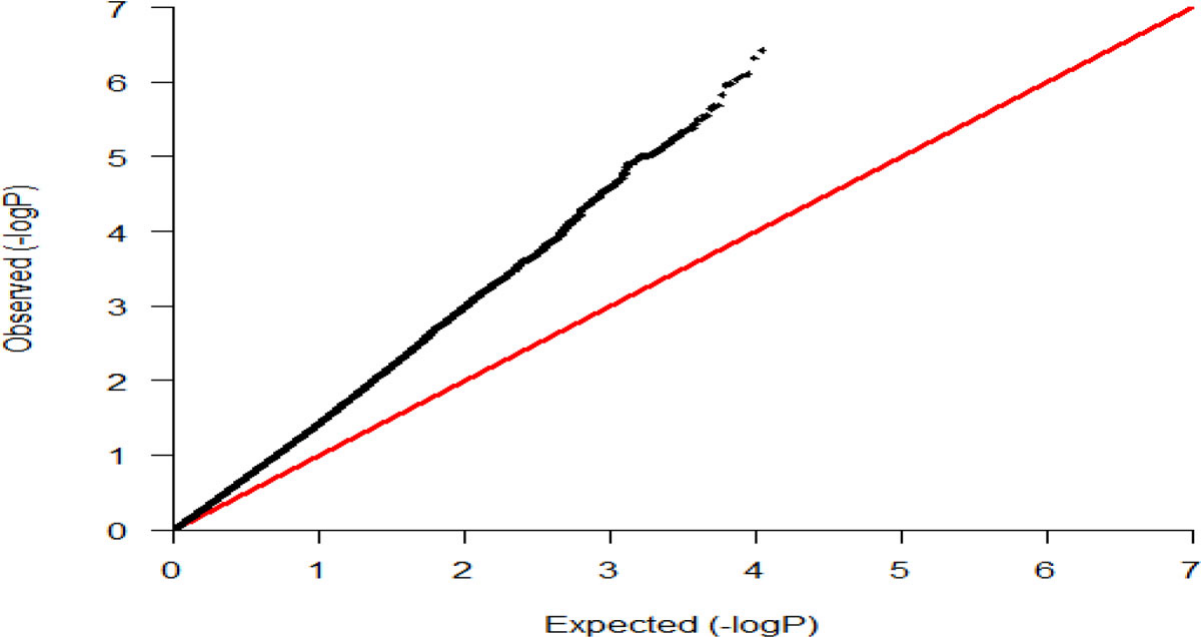
**Quantile-quantile (Q-Q) plot of joint association *p *values for variants on chromosomes 3 and 9**.

Table 1 displays the results of the bivariate linear mixed model analysis using F-test for the first 15 most significant SNPs in each of chromosomes 3 and 9. The Manhattan plot and quantile-quantile (Q-Q) plot of the joint association *p *values for all SNPs are shown in Figures 1 and 2, respectively. The joint association *p *values using $\chi_{2}^{2}$ test statistic were in general slightly smaller but very similar to that from F statistic (the difference was very small for the most significant SNPs, for instance, *p *value by $\chi_{2}^{2}$ for *rs9632874 *was smaller by 1.98E-10 [results not shown]). Three SNPs, *rs12634258, rs7647249, rs4533619 *from intergenic region on chromosome 3, and 4 SNPs *rs9632874, rs12335766, rs10122040 *from gene *TTC39B *and *rs7864652 *from gene *BNC2 *on chromosome 9, appeared to be significant at genome-wide level.

**Table 1:** Top 15 most significant SNPs in the bivariate linear mixed model analysis.

Chromosome 3						Chromosome 9					
**SNP**	**MiA**	**MAF**	**GENE**	**BP**	**P**	**SNP**	**MiA**	**MAF**	**GENE**	**BP**	**P**

rs12634258	T	0.38		61291738	1.54E-09	rs9632874	C	0.08	*TTC39B*	15270875	2.40E-11

rs7647249	C	0.20		133245961	2.70E-08	rs7864652	T	0.07	*BNC2*	16456759	1.39E-09

rs4533619	C	0.26		42289812	5.10E-08	rs12335766	G	0.08	*TTC39B*	15277123	8.54E-09

rs748191	G	0.19		5888909	3.78E-07	rs10122040	T	0.12	*TTC39B*	15279214	1.28E-08

rs1821942	G	0.41	*FHIT*	61116009	8.30E-07	rs4879586	C	0.36	*ACO1*	32427874	4.78E-07

rs9311317	G	0.30		42295806	1.49E-06	rs7034380	G	0.44	*FREM1*	14908056	7.73E-07

rs4681514	G	0.05	*TM4SF4*	149211660	2.84E-06	rs10858108	G	0.22		138337411	9.26E-07

rs12714954	A	0.24	*LOC339862*	18303505	2.87E-06	rs10858106	G	0.22		138332424	1.06E-06

rs17634797	A	0.05	*FHIT*	61014061	3.16E-06	rs10122098	A	0.11		12918226	1.10E-06

rs13071249	A	0.06	*LOC339862*	18174701	3.23E-06	rs7022225	G	0.32	*PALM2*	112536455	2.06E-06

rs9850400	C	0.44	*FHIT*	61101006	3.68E-06	rs7038509	T	0.32	*PALM2*	112536525	2.06E-06

rs1439008	T	0.48	*FHIT*	61066987	4.12E-06	rs10961479	T	0.06	*NFIB*	14332760	2.32E-06

rs10936111	A	0.38		157586847	5.60E-06	rs9299075	A	0.26	*PTPRD*	8798347	4.19E-06

rs1439004	A	0.41	*FHIT*	61103897	6.09E-06	rs10817738	T	0.20	*DEC1*	118029729	4.37E-06

rs6806415	C	0.41	*FHIT*	61103609	6.09E-06	rs2809247	A	0.28	*AK8*	135654476	4.48E-06

BP, Base pair position; MAF, minor allele frequency; MiA, minor allele; P, joint association p value; SNP, single-nucleotide polymorphism.

### Simulation results

The bivariate linear mixed model analysis had 76.5% power to detect the effect of *rs6442089 *jointly on SBP and DBP; whereas the separate univariate linear mixed model analyses had only 30.5% and 45.0% power to detect of effects of the same SNP on SBP and DBP, respectively at *α *= 0.05, using 141 unrelated subjects. At *α *= 7.2 × 10^−8 ^the association of SNP was detected in 5 (2.5%) of simulated data sets in the bivariate analysis, but the corresponding univariate analysis did not detect the association with either of the phenotype in any simulated data set. The univariate analysis produced the smallest *p *value of 1.6 × 10^−5 ^for SBP and that for DBP was slightly bigger (Table 2), whereas the bivariate analysis resulted in smaller *p *values in 14.5% of the simulated data sets.

**Table 2:** Top 3 *p *values and corresponding simulation data sets from bivariate and separate univariate mixed model analyses, and *p *values from other methods in the same simulation data sets.

Analysis type	*p *Value rank	Simulation data set #	*p *Value		
			
			Bivariate	SBP	DBP
Bivariate model for both SBP and DBP	1	57	2.2E-10	0.000016159	0.0702504382
	
	2	1	8.2E-09	0.0206245995	0.0008121652
	
	3	142	9.5E-09	0.0009235493	0.0003764385

Univariate model for SBP only	1	57	2.2E-10	0.000016159	0.0702504382
	
	2	105	3.5E-07	0.0001905345	0.0357532908
	
	3	56	7.5E-08	0.0002211798	0.1136335665

Univariate model for DBP only	1	35	2.0E-06	0.0122982922	0.0001930709
	
	2	41	1.4E-07	0.0064351761	0.0002340975
	
	3	142	9.5E-09	0.0009235493	0.0003764385

## Discussion

In bivariate linear mixed model analysis, we observed associations of a few SNPs from intergenic regions and *TTC39B *gene with high blood pressure despite the small sample size. The bivariate mixed model framework to the GAW18 simulated data suggested that the bivariate analysis is more powerful than univariate approach to analyze longitudinal data when phenotypes are correlated. An earlier simulation study [8] also found that bivariate approach is in general more powerful than the univariate analysis when quantitative traits are correlated. High blood pressure is believed to be influenced by hundreds of genes with generally very small to modest effects. So we need statistical method with improved power to detect such associations and bivariate method to longitudinal data can be a useful strategy to identify the list of SNPs that can be followed up for replication or validation of their associations with the phenotypes of interest.

However, we wish to underscore the limitation that our simulation study was not extensive; we just compared the power of the bivariate approach with that of the univariate approach for only 1 SNP. We did not assess the type I error rate of the bivariate method, which could be inflated, especially for a small data set. In fact, the Q-Q plot (see Figure 2) of the joint association *p *values (see Figure 2) strongly suggests such inflation of the error rate, although the deviation from null distribution could also be an indication of the presence of population substructure or admixture. Also, one needs to be cautious in interpreting our results from the real data analysis as we have a number of limitations in this study, including small sample size, missing observations, uncertainty about the underlying genetic model, selection of an appropriate correlation structure, random effect assumptions, and choice of test statistic. For instance, although we chose a covariance (correlation) structure between *AR*(1) and *UN, AR*(1) had a similar or somewhat smaller AIC. However, it assumes that measurements were made at an equal interval over time for each and all phenotypes [4,5]. But this was an unrealistic assumption in our data because the time interval between the first and second examination ranged from 1.4 to 7.6 years. Next, it also assumes the same correlation between measurements of 2 phenotypes at a time and between any 2 measurements of all the phenotypes for all subjects, which might not be true. Although it involves estimating more parameters, a *UN *assumption is more flexible, consequently, we employed it our bivariate analysis. A detail investigation of the properties of bivariate method in many realistic scenarios via extensive simulation is warranted before we draw a general conclusion about the usefulness of the bivariate method for the correlated repeatedly measured phenotypes.

## Conclusion

A bivariate approach to test associations of genetic variants with multiple phenotypes jointly measured over time from the same individuals could be a useful strategy to identify genetic variants that deserve further investigation as it can exploit the correlation structure between phenotypes at a time and the same phenotype over time.

## Competing interests

The authors declare that they have no competing interests.

## Authors' contributions

JB and BN designed the analysis plan. BN conducted the statistical analyses, and drafted the manuscript. JB assisted in drafting, and critically revised the manuscript. Both authors read and approved the final manuscript.
